# A septo-temporal molecular gradient of *sfrp3* in the dentate gyrus differentially regulates quiescent adult hippocampal neural stem cell activation

**DOI:** 10.1186/s13041-015-0143-9

**Published:** 2015-09-04

**Authors:** Jiaqi Sun, Michael A. Bonaguidi, Heechul Jun, Junjie U. Guo, Gerald J. Sun, Brett Will, Zhengang Yang, Mi-Hyeon Jang, Hongjun Song, Guo-li Ming, Kimberly M. Christian

**Affiliations:** Tsinghua-Peking Joint Center for Life Sciences, Tsinghua University, Beijing, 100084 P.R. China; Institute for Cell Engineering, Johns Hopkins University School of Medicine, Baltimore, MD 21205 USA; Department of Neurology, Johns Hopkins University School of Medicine, Baltimore, MD 21205 USA; Department of Neurologic Surgery, Department of Biochemistry and Molecular Biology, Mayo Clinic College of Medicine, Rochester, MN 55905 USA; The Solomon H. Snyder Department of Neuroscience, Johns Hopkins University School of Medicine, Baltimore, MD 21205 USA; Institutes of Brain Science and State Key Laboratory of Medical Neurobiology, Fudan University, Shanghai, 200032 China; Department of Psychiatry and Behavioral Sciences, Johns Hopkins University School of Medicine, Baltimore, MD 21205 USA; Present Address: Broad CIRM Center and Department of Stem Cell Biology and Regenerative Medicine, Zilkha Neurogenetic Institute, University of Southern California Keck School of Medicine, Los Angeles, CA 90033 USA; Present Address: Whitehead Institute for Biomedical Research, Cambridge, MA 02142 USA

**Keywords:** Adult hippocampal neurogenesis, Molecular gradient, neural stem cells, Septo-temporal axis, Wnt, niche

## Abstract

**Background:**

A converging body of evidence indicates that levels of adult hippocampal neurogenesis vary along the septo-temporal axis of the dentate gyrus, but the molecular mechanisms underlying this regional heterogeneity are not known. We previously identified a niche mechanism regulating proliferation and neuronal development in the adult mouse dentate gyrus resulting from the activity-regulated expression of *secreted frizzled-related protein 3* (*sfrp3*) by mature neurons, which suppresses activation of radial glia-like neural stem cells (RGLs) through inhibition of Wingless/INT (WNT) protein signaling.

**Results:**

Here, we show that activation rates within the quiescent RGL population decrease gradually along the septo-temporal axis in the adult mouse dentate gyrus, as defined by MCM2 expression in RGLs. Using *in situ* hybridization and quantitative real-time PCR, we identified an inverse septal-to-temporal increase in the expression of *sfrp3* that emerges during postnatal development. Elimination of *sfrp3* and its molecular gradient leads to increased RGL activation, preferentially in the temporal region of the adult dentate gyrus.

**Conclusions:**

Our study identifies a niche mechanism that contributes to the graded distribution of neurogenesis in the adult dentate gyrus and has important implications for understanding functional differences associated with adult hippocampal neurogenesis along the septo-temporal axis.

## Background

Along with its well-known role in learning and memory, the hippocampus is a neural structure involved in the regulation of motivational behaviors, emotional states and stress responses [1, 2]. The rodent hippocampus is an elongated, laminated structure with its long axis extending rostro-dorsally from the septal nuclei of the basal forebrain, over and behind the thalamus, and then caudo-ventrally to the temporal lobe. This longitudinal axis of the hippocampus is usually referred to as the septo-temporal (or dorso-ventral) axis [3, 4]. Differences in anatomical connections and electrophysiological properties along the septo-temporal axis of the hippocampus have been well documented [5–11]. In addition, studies using targeted lesions and inactivation have revealed functional differences along this hippocampal axis. Specifically, the septal (dorsal) portion appears to be preferentially engaged in learning and memory processes associated with navigation and exploration, while the temporal (ventral) hippocampus appears to be more involved in mood and anxiety-related behaviors [12, 13]. Consistent with these findings, a recent study has demonstrated that acute activation of granule cells specifically in the dorsal or ventral hippocampal dentate gyrus differentially suppresses contextual learning or innate anxiety, respectively [14]. Moreover, region-specific gene expression supports segregation of the hippocampus into septal, intermediate and temporal zones [11, 15].

The adult mammalian hippocampus continuously generates new neurons that integrate into preexisting neuronal networks [16–18]. Adult neurogenesis is a complex process whereby quiescent radial glia-like neural stem cells (RGLs) give rise to newborn neurons that mature over several weeks [16–19]. Cumulative evidence suggests that several properties of adult neurogenesis vary throughout the longitudinal (septo-temporal) axis of the hippocampus. The magnitude of adult hippocampal neurogenesis is lower in the temporal region, as assessed by the number of RGLs, intermediate progenitor cells and immature neurons [20, 21]. The pace of neurogenesis is also slower in the temporal region, but it appears to be more responsive to local niche dynamics and exogenous factors [22]. Not only can changes in neuronal activity alter the tempo of newborn neuron maturation more dramatically, but the age-related decline in precursor number occurs more rapidly in the temporal dentate gyrus [22–24]. Furthermore, studies indicate a functional dissociation for adult hippocampal neurogenesis along the longitudinal axis wherein newborn neurons in the dorsal dentate gyrus modulate learning of contextual discrimination under some conditions, while immature neurons in the ventral dentate gyrus mediate anxiolytic effects of the anti-depressant fluoxetine [25]. How these differences in neurogenesis along the longitudinal axis of hippocampus are regulated is a fundamental question that remains to be answered.

Morphogens and their expression gradients play an essential role in the patterning of organogenesis during embryonic development [26]. Among these, Wingless/INT (Wnt) family members are critical morphogens that regulate numerous developmental processes, including neural stem cell maintenance and differentiation in the vertebrate central nervous system (CNS) [27, 28]. During embryonic and early postnatal development, Wnt signaling is essential for the proper formation of the hippocampus. For example, Wnt3a is expressed in the cortical hem, which serves as a signaling center for hippocampal development at the tip of the caudomedial cortical wall, and is crucial for the normal growth of the hippocampus [29]. Furthermore, fate specification is determined by the relative strength of Wnt signaling such that strong Wnt signaling biases differentiation toward a dentate granule cell fate, while moderate Wnt signaling specifies pyramidal cells of the cornus ammonis (CA) layers [30]. In the dentate gyrus of the adult mouse hippocampus, many Wnts and their inhibitors are expressed [31] and functional studies have revealed that Wnt signaling regulates multiple steps of adult hippocampal neurogenesis under different physiological conditions [32–39]. In particular, we recently showed that mature granule cells in the adult mouse dentate gyrus express secreted Frizzled-related protein 3 (sfrp3), a secreted inhibitor of Wnt signaling, and that deletion of *sfrp3* leads to increased activation of quiescent RGLs and production of new neurons [32], as well as behaviors mimicking those observed with long-term antidepressant treatment [40]. In this study, we examined whether *sfrp3* could contribute to differences in the magnitude of adult hippocampal neurogenesis along the septo-temporal axis.

## Results

### Heterogeneity of quiescent RGL activation along the septo-temporal axis of adult mouse dentate gyrus

**Fig. 1:**
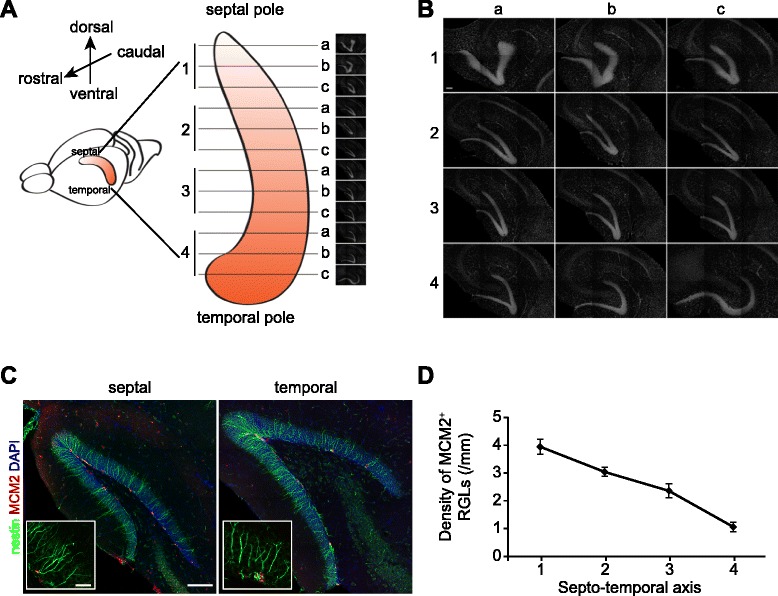
Activation of quiescent radial glia-like neural stem cells (RGLs) along the septo-temporal axis of the adult hippocampus. **a** A schematic view of the mouse brain depicting the three-dimensional structure of the hippocampus and the orientation of the sections analyzed in this study. **b** Representative images of sections used for the analysis from septal (1a) to temporal (4c). The sections were stained with DAPI (*grey*). Scale bar, 100 μm. **c** Representative confocal images of immunostaining of MCM2 (*red*), nestin (*green*) and DAPI staining (*blue*) in the septal (*left panel*) and temporal (*right panel*) dentate gyrus of the same mouse. Inset, higher magnification views. Scale bars: 100 μm; inset, 25 μm. **d** Quantitative analysis of MCM2^+^nestin^+^ RGLs along the septo-temporal axis. Horizontal axis labels correspond to the numbers in Fig. 1a. Values represent mean ± S.E.M (*n* = 4)

Due to the curvature and orientation of the hippocampus (Fig. 1a), sectioning the brain in one of the three traditional planes (coronal, horizontal, sagittal) produces sections in which the septo-temporal coordinates are hard to define. To better visualize adult hippocampal neurogenesis along the septo-temporal axis, we first established a new method for sectioning. We fixed hippocampi dissected from the adult mouse brain and embedded them into O.C.T. mounting solution. The individual hippocampus was positioned such that coronal-like sections were obtained from much of the septo-temporal extent of the structure (Fig. 1a-b). For quantification, we divided the hippocampus into four regions along the septo-temporal axis (Fig. 1a-b). Using this approach, we first investigated whether a difference in the activation of quiescent RGLs exists along the septo-temporal axis of the hippocampus of adult mice. We identified RGLs as nestin^+^ precursors localized within the sub-granular zone (SGZ) with radial processes extending towards the molecular layer [41] (Fig. 1c). We further used the expression of minichromosome maintenance complex component 2 (MCM2) as a marker for activation of largely quiescent RGLs since thymidine analogs such as EdU and BrdU are only incorporated during the S phase of cell cycle and preferentially label intermediate progenitors and neuroblasts with a single injection [41–46] (Fig. 1c). Stereological quantification along the SGZ in four regions showed a gradual decrease in the density of MCM2^+^ RGLs along the septo-temporal axis of the hippocampus (Fig. 1c-d). These results confirmed previous findings of septo-temporal differences in adult hippocampal neurogenesis [20] and further identified a clear gradient in which activation of quiescent adult neural stem cells decreases along the septo-temporal axis.

### A gradient of *sfrp3* expression along the septo-temporal axis of the adult dentate gyrus

**Fig. 2:**
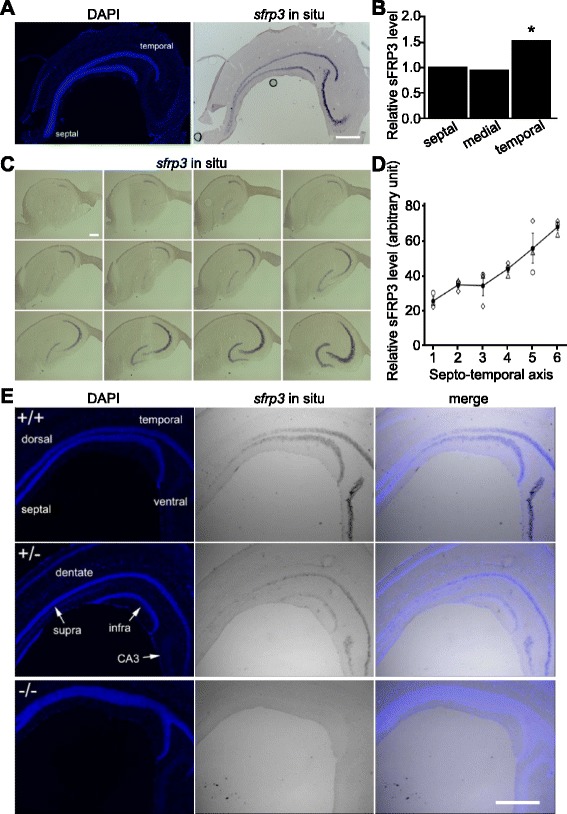
A gradient of *sfrp3* expression in the dentate gyrus of adult mouse hippocampus. **a** Mid-sagittal section of dissected mouse hippocampus showing *in situ* hybridization (ISH) for *sfrp3* mRNA. Scale bar, 500 μm. **b** Quantitative analysis of *sfrp3* expression by quantitative real-time PCR. Values present mean ± S.E.M. (*n* = 4; * < 0.05; ANOVA). **c**. Representative images of ISH for *sfrp3* mRNA on twelve serial sections along the septo-temporal axis, with the planes oriented as shown in Fig. 1a. Scale bar, 10 μm. **d** Quantification of signal intensity for *sfrp3 in situ*, which was performed on the images shown in Fig. 2c. *Open circles, diamonds* and *triangles* represent sfrp3 mRNA from septo-temporal axis of three individual mice. Closed circles are the mean of the three mice. Values represent mean ± S.E.M. (*n* = 3). **e** Representative images of *sfrp3* mRNA *in situ* in the dentate gyrus of adult *sfrp3* knockout (^−/−^), heterozygous (^+/−^), and wild-type (^+/+^) littermates. Scale bar, 500 μm

Next, we investigated the potential molecular niche mechanism underlying this heterogeneity in neurogenesis along the septo-temporal axis of the adult hippocampus. Several studies have revealed specific molecular domains and regionalization within the dentate gyrus [11, 15]. We focused on *sfrp3* based on several of our recent findings. First, we found that in adult mice *sfrp3* is highly expressed in mature dentate granule cells and suppresses activation of quiescent RGLs and inhibits maturation of immature neurons [32]. Second, *sfrp3* expression is downregulated by various antidepressant treatments, including electroconvulsive treatment and chemical antidepressants, whereas *sfrp3* knockout mice exhibit behaviors resembling those following antidepressant treatment [40]. Third, we found that three single-nucleotide polymorphisms (SNPs) in the human *SFRP3* gene are significantly associated with early antidepressant responses in a human patient cohort [40]. Given the proposed functional differentiation of the hippocampus along its long axis, we assessed whether the expression pattern of *sfrp3* aligns with this dissociation at the molecular level. We examined *sfrp3* expression along the longitudinal axis of the adult dentate gyrus using *in situ* hybridization and quantitative real-time PCR analyses. To directly visualize *sfrp3* expression along the septo-temporal axis in a single section, we developed another new approach to section the dissected hippocampus in the sagittal plane (Fig. 2a). Consistent with previous reports [32, 47], *sfrp3* expression was enriched in the granule layer of the adult dentate gyrus (Fig. 2a). Interestingly, the *sfrp3 in situ* signal intensity increased along the longitudinal axis of adult hippocampus from the septal pole to the temporal pole (Fig. 2a). Quantitative real-time PCR in samples of micro-dissected dentate gyrus further confirmed higher *sfrp3* expression in the temporal dentate gyrus (Fig. 2b). Notably, *in situ* analysis showed that the *sfrp3* gradient appeared to be continuous across the septo-temporal axis of the hippocampus without sharp step changes suggestive of “border” transitions (Fig. 2a). We confirmed this result with densitometric measurements of *in situ* signal intensity on coronal sections of dissected hippocampus along the entire axis of hippocampus (Fig. 2c-d). To confirm the specificity of the *sfrp3 in situ* probe used, we compared wild-type (+/+), heterozygous (+/−) and knockout (−/−) *sfrp3* animals. Indeed, no signal was observed in knockout animals in sections processed in parallel with other genotypes (Fig. 2e). Interestingly, a gradient was present in the heterozygous mice, although the expression level appeared to be reduced by ~50 % (Fig. 2e). Together, these results demonstrated that *sfrp3* expression in the adult mouse dentate gyrus exhibits a smooth gradient with increasing expression along the septo-temporal axis.

### Developmental establishment of the *sfrp3* expression gradient in the dentate gyrus

**Fig. 3:**
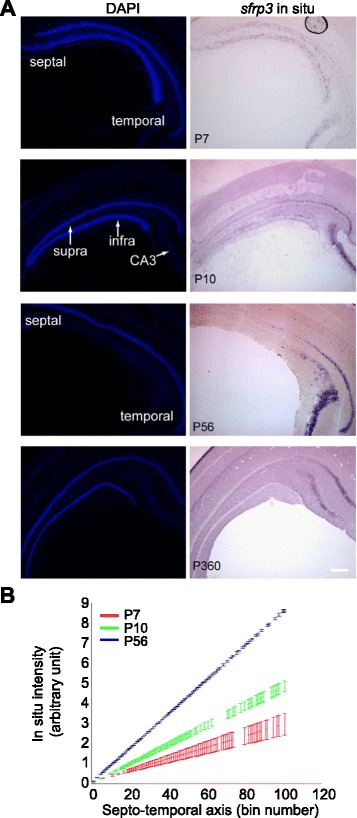
A gradient of *sfrp3* expression in dentate gyrus from early postnatal to aged animals. **a** Representative images of *sfrp3* mRNA *in situ* in the dentate gyrus of wildtype mice at different ages (P7, P10, P56 and P360). Scale bar, 250 μm. **b** Quantification of signal intensity for *sfrp3 in situ*, which was performed on the images shown in Fig. 3a. Reconstructed dentate gyri from sagittal sections were divided into 100 bins (represented on the X axis) for quantification of signal intensity along the septo-temporal axis. Values represent mean ± S.E.M. (*n* = 3). The *sfrp3* mRNA expression increases from septal to temporal dentate gyrus at each age from P7 to P56 in WT mice with an increase in slope during the interval of P7-P56

Although it is well-known that morphogens act as graded positional cues to control cell fate specification in many developing tissues, the presence of a gradient in adult tissue is very rare. We next investigated the developmental time point when the gradient expression pattern of *sfrp3* is established. Previous studies have examined *sfrp3* expression during early embryonic development. Specifically, *sfrp3* is not expressed in the cortical hem, which is the hippocampal organizer [48], but is instead expressed dorsal to this region at E14.5 and becomes confined to the dentate gyrus at E17.5 [49]. Using *in situ* hybridization, we examined the magnitude and time course of *sfrp3* expression from postnatal day 7 (P7), when the dentate gyrus is recognizable as a morphological structure with two blades of discrete dentate granule cell layers (Fig. 3a). At P7, the *sfrp3 in situ* signal was scattered in the dentate granule cell layer and CA3. By P10, *sfrp3* expression became prominent in the dentate gyrus, although still weaker than in the adult dentate gyrus. The gradient expression pattern was observed at P7, became readily apparent by P10, and persisted at P360 (Fig. 3a). Quantitative analysis showed that the slope of the *sfrp3* mRNA expression gradient increased between P7 and P56 (Fig. 3b). Together, these results revealed that the gradient expression pattern of *sfrp3* is initiated during early postnatal stages and becomes more pronounced later and maintained during adulthood.

### Functional impact of *sfpr3* gradient on neurogenesis along the septo-temporal axis

**Fig. 4:**
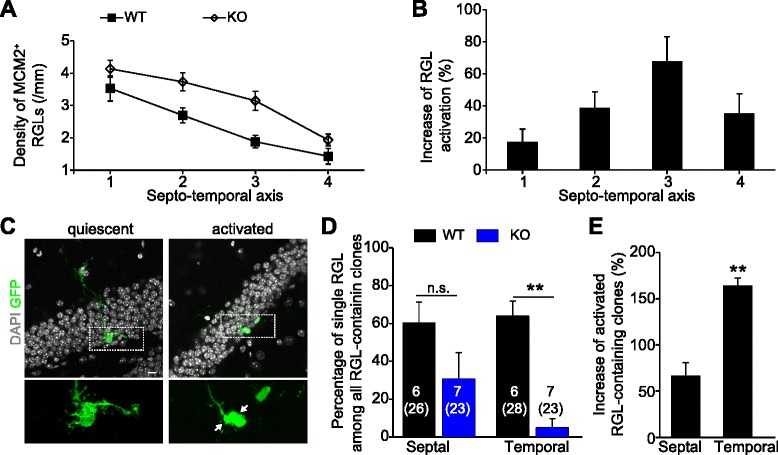
Activation of quiescent radial glia-like neural stem cells increases in adult *sfrp3* knockout (KO) mice. **a** Quantitative analysis of MCM2^+^nestin^+^ RGLs along the septo-temporal axis of adult *sfrp3* KO and wildtype (WT) littermates. Values present mean ± S.E.M. [*n* = 6 (KO), 7 (WT)]. **b** Higher relative increase in RGL activation in the temporal dentate gyrus of adult *sfrp3* KO mice. Same groups of animals in Fig. 4b were analyzed. Data were normalized to control WT group from septal (1) to temporal (4) dentate gyrus. **c** Representative confocal images of immunostaining of GFP (*green*) for quiescent and activated clones in the adult *nestin-CreER* ^*T2+/−*^ *;Z/EG* ^*f/+*^ *; sfrp3* ^*−/−*^ KO mice and *nestin-CreER* ^*T2+/−*^ *;Z/EG* ^*f/+*^ *; sfrp3* ^*+/+*^ control mice. Scale bar = 10 μm. **d** Quantification of increased RGL activation at both septal and temporal poles in the dentate gyrus of adult *sfrp3* KO mice examined at 7 dpi. Numbers associated with the bar graph indicate the number of dentate gyri and total number of clones (in parentheses) analyzed, *grey bars* = WT, *blue bars* = KO. Values represent mean ± S.E.M. (*P < 0.01; n.s. *P* > 0.1; AVOVA). **e** Stronger relative increase of RGL activation in the temporal dentate gryus of adult *sfrp3* KO mice. Same groups of animals in Fig. 4d were analyzed. Data were normalized to control WT group

Finally, we examined the contribution of the *sfrp3* molecular gradient on the differential regulation of quiescent RGL activation along the septo-temporal axis. Although it is impossible to rule out compensatory effects during development from germline transgenic mice, our previous analyses of adult *sfrp3* knockout mice did not indicate any gross or cumulative morphological effects on the dentate gyrus [40]. Consistent with our previous population study, *sfrp3* knockout mice exhibited increased activation of quiescent RGLs as shown by the increased density of MCM2^+^ RGLs (Fig. 4a). This increase occurred in both septal and temporal regions and, notably, the magnitude of the change in quiescent RGL activation gradually increased from regions 1 to 3 along the septo-temporal axis, but not in region 4 at the most temporal pole (Fig. 4b). Therefore, removal of *sfrp3* expression and its associated gradient dampens, but does not completely abolish, the septo-temporal differences in quiescent RGL activation during adult hippocampal neurogenesis. These results support an active role for the *sfrp3* gradient in differential regulation of stem cell activation along the septo-temporal axis, but also point to additional mechanism(s) that may mediate residual differences, particularly in the temporal pole.

To further support our model, we examined quiescent RGL activation using an independent approach via clonal lineage-tracing of individual quiescent RGLs. We previously established a clonal assay in which we could label individual quiescent RGLs in adult *nestin-CreER*^*T2+/−*^*;Z/EG*^*f/+*^ mice with injection of a single low dose of tamoxifen to quantify their activation rates and fate choices [32, 41, 45]. We generated adult *nestin-CreER*^*T2+/−*^*;Z/EG*^*f/+*^*; sfrp3*^*−/−*^and *nestin-CreER*^*T2+/−*^*;Z/EG*^*f/+*^*; sfrp3*^*+/+*^ mice and performed short-term clonal analysis. We quantified quiescent (an isolated RGL) or activated (an RGL with one or more progeny in close proximity) individual clones with respect to their septal or temporal localization in the dentate gyrus (Fig. 4c), which indicated the cumulative RGL activation during the testing period. Consistent with previous studies [32], *sfrp3* knockout mice exhibited an increase in the number of activated RGLs throughout the hippocampus (Fig. 4d). Notably, the increase in RGL activation is more than doubled in the temporal region compared to that in the septal region (Fig. 4e). These results also support our model that the graded *sfrp3* expression along the longitudinal hippocampal axis contributes to the regional differences in RGL activation rates.

## Discussion

In the developing brain, morphogen gradients are commonly used to pattern neurogenic regions into distinct functional domains. Examples of such gradients are WNTs that are responsible for specifying dorsal fates during telencephalic neurogenesis [50]. Several members of the Wnt family (including Wnt-2b, Wnt-3a, Wnt-5a, Wnt-7b, and Wnt-8b), as well as members of the SFRP family of Wnt inhibitors, are highly expressed in the cortical hem, a medial telencephalic signaling center, which is also the primary hippocampal organizer [28, 51]. Gradients of Wnt family members are well studied in the developing hippocampus [49]. Whether any of these gradients persist into adulthood was not previously known. Early in development, *sfrp3* is expressed weakly in the medial neuroepithelium and medial non-neural mesenchyme, but is excluded from the cortical hem. Later, *sfrp3* is expressed in cells from the dentate notch to the developing dentate gyrus along the migratory route of newly born dentate granule cells and their precursors. In addition, *sfrp3* is expressed in the tertiary matrix, which is the displaced mitotic zone that transiently forms at the termination of this migratory route in the hilus of the dentate gyrus [49]. Here we identified a graded expression pattern of *sfrp3* that emerges by postnatal day 7. Our study revealed, for the first time, that a morphogen-related protein gradient is generated postnatally and maintained in the adult mammalian brain.

The proliferation and integration of adult-born neurons into existing hippocampal circuitry has been implicated in a wide range of behaviors, including context discrimination, pattern separation, spatial learning, anxiety, and responses to antidepressants [18, 52–57]. These diverse functions may reflect differences in the local hippocampal network along the septal-temporal axis, with respect to anatomical connections and electrophysiological properties. For example, serotonergic fibers provide denser input to the temporal hippocampus with a concomitant enrichment of 5-HT1A and 2C receptors [10, 58]. Long-term potentiation (LTP) is both larger and longer lasting in septal slices [59]. Growing evidence supports that adult neurogenesis in the dentate gyrus is also heterogeneous along this longitudinal axis. A higher number of proliferating cells and faster maturation rates occur in the septal dentate gyrus compared to the temporal region [24]. Because adult neurogenesis has been implicated in both cognitive and affective behaviors, an exciting possibility is that adult-born granule cells in the septal and temporal hippocampus may be functionally dissociated [25]. However, little was known about potential niche mechanisms that could contribute to septo-temporal heterogeneity in hippocampal neurogenesis processes and properties. Our study identifies a gradient of expression of *sfrp3* (inferred from its mRNA levels), a niche factor secreted by mature dentate granule neurons, that results in differential control of neurogenesis along the longitudinal axis of the dentate gyrus. Both population analysis and in vivo clonal analysis, using a genetic sparse-labeling approach, indicate that removal of *sfrp3* and its gradient result in preferentially increased RGL activation in the temporal dentate gyrus. Since higher levels of *sfrp3* are expressed under physiological conditions in this region, our results functionally link neural stem cell activity to specific patterns of gene expression.

Our previous study identified *sfrp3* as a neuronal activity-regulated niche factor that exhibits control over multiple steps of adult hippocampal neurogenesis, including progenitor proliferation, newborn neuron maturation, dendritic growth and dendritic spine formation, as well as the activation of quiescent adult neural stem cells [32]. *Sfrp3* is an essential mediator of some antidepressant actions in animal models and polymorphisms in the human gene are significantly associated with partial antidepressant responses in patients, which suggest that manipulation of SFRP3 action may represent a novel therapeutic approach to treat depression [40]. Here, we reveal higher expression levels of *sfrp3* in the temporal hippocampus, which is the region more actively involved in mediating affective behaviors. Our study thus suggests how SFRP3 could be an efficacious and highly restricted extracellular target for fine-tuning adult hippocampal neurogenesis in a region-specific manner. Taken together, our results reveal a molecular gradient generated by local mature dentate granule neurons that couples adult neurogenesis to heterogeneities in the surrounding niche. This gradient provides a mechanism that may contribute to regional differences in adult neurogenesis and parallels the functional differences along the septo-temporal axis. As several genes have recently been reported to be differentially expressed and regulated along the septo-temporal axis of the hippocampus [11, 15], *sfrp3* may be one of multiple molecular gradients contributing to heterogeneous functional properties of the hippocampus through regulation of neural stem cell activation, neuronal development and circuit integration during adult neurogenesis.

## Conclusions

We found that *sfrp3*, a secreted inhibitor of Wnt signaling, is expressed in a gradient along the septo-temporal axis of the dentate gyrus that is established during postnatal development. We provide functional evidence that this molecular gradient regulates quiescent RGL activation. Our study thus demonstrates, for the first time, a molecular niche mechanism to support region-specific differences in adult hippocampal neurogenesis along the longitudinal axis of hippocampus.

## Materials and methods

### Animals, housing, administration of tamoxifen and tissue processing

Eight-week-old mice of the following genotypes were used: wild-type (C57BL/6), *sfrp3* wild-type (WT) and knockout (KO) (B6;129), *nestin-CreER*^*T2+/−*^*;Z/EG*^*f/+*^*; sfrp3*^*−/−*^ (C57BL/6) and *nestin-CreER*^*T2+/−*^*;Z/EG*^*f/+*^*; sfrp3*^*+/+*^ (C57BL/6). Animals were housed in the standard facility. All animal procedures used in this study were performed in accordance with the protocol approved by the Institutional Animal Care and Use Committee.

A single low dose of tamoxifen (62 mg/kg body weight, i.p.; Sigma) in 2-month-old mice resulted in sparse labeling at the clonal level for analysis at 7 dpi in both WT and *sfrp3* KO mice, as previously described [41]. Mice were anaesthetized (100 μg ketamine, 10 μg xylazine in 10 μl saline per gram) and perfused with 4 % paraformaldehyde (PFA) and brains were postfixed in PFA overnight. Brains were then transferred to 30 % sucrose and stored at 4 °C until brains sank to the bottom of tube.

### Anatomical definitions and sectioning

For analysis of neurogenesis along the longitudinal axis, the right hippocampus was dissected and sectioned perpendicular to its long axis to enable comparable analyses along the entire axis. This axis is most precisely described as the septo-temporal axis (Fig. 1a). Sections were cut at 40 μm using a cryostat (Leica CM 3050S) for a total of ~80 sections. Every sixth section was collected and processed for immunostaining.

For clonal analysis, coronal brain sections (40 μm) through the entire dentate gyrus were collected in a serial order for a total of ~50 sections using a microtome (Leica SM 2010R), which is along the anterior-posterior axis.

### Immunostaining, confocal imaging, *in situ* hybridization and quantitative real-time reverse transcription PCR

For immunostaining with anti-nestin and anti-MCM2, an antigen retrieval protocol was performed by microwaving sections in boiled citric buffer for 7 min as described previously [41]. Immunostaining was performed with the following primary antibodies: anti-GFP (Rockland; goat; 1:500 dilution), anti-nestin (Aves; chick; 1:500 dilution), anti-MCM2 (BD; mouse; 1:500 dilution). Images were acquired on a Zeiss LSM 710 confocal system (Carl Zeiss) with a × 40 objective lens using a multitrack configuration.

*In situ* hybridization analysis was performed similarly as previously described [60]. Briefly, 4 % paraformamide-fixed cryo-protected brain tissue samples were embedded in O.C.T. mounting solution and frozen at −80 °C. Brain sections (20 μm) were cut onto Superfrost-Plus slides (Fisher Scientific). Full-length digoxygenin-labeled antisense riboprobe for *sfrp3* was prepared by *in vitro* transcription. Sections were hybridized with the riboprobes at 65 °C overnight, and washed once in 5X SSC and 1 % SDS, then twice in 2X SSC without SDS for 30 min each at 65 °C. After overnight incubation with alkaline phosphatase-conjugated anti-digoxygenin antibody at 4 °C, hybridized riboprobes were visualized using nitro blue tetrazolium (NBT, 35 μg/ml)/5-bromo-4-chloro-3-indolyl phosphate (BCIP, 18 μg/ml) color reaction at room temperature.

For quantitative real-time reverse transcription PCR, dentate gyrus tissue was rapidly micro-dissected from adult WT mice and cut into three fragments (septal, intermediate and temporal regions). For gene expression analysis, the total RNA fraction was immediately isolated after dissection (Qiagen), treated with DNAase and reverse-transcribed into the first-strand cDNA (Invitrogen). Specific primers as followed were used in SYBR-green based quantitative real-time PCR to measure the expression level of target genes with the ∆∆Ct method (ABI).

GAPDH: 5’- GTATTGGGCGCCTGGTCACC-3’ (forward), 5’- CGCTCCTGGAAGATGGTGATGG-3’ (reverse); sFRP3: 5’- CAAGGGACACCGTCAATCTT-3’ (forward), 5’- CATATCCCAGCGCTTGACTT-3’ (reverse).

### Quantification and statistical analysis

For quantification of signal intensity of *in situ* hybridization on hippocampal coronal sections, brightfield images were taken at the same exposure and converted into grey scale. Granule cell layers of dentate gyrus were circled and mean grey values were obtained. For normalization, an area without signal was selected and mean grey values were calculated. The intensity indicated by the mean grey values in the granule cell layer after subtracting the mean grey value in the area without signal represents the relative expression levels. For quantification of signal intensity of *in situ* hybridization in hippocampal sagittal sections, brightfield images were manually segmented in LSM image browser (Zeiss) using the 'closed free shape curved drawing' and the 'extract region' tools. Segmented dentate gyri were then manually aligned using Reconstruct [45] and rotated such that the dentate gyrus occupied a maximum horizontal space. Aligned images were loaded as matrices into Matlab (Mathworks) for quantitative measurements. Matrices were summed across rows and the resulting vector was divided into 100 bins. The mean value for each bin was calculated and the resulting 100 mean values were used as the quantitative expression data across the dentate gyrus for each analyzed image.

For quantification of MCM2^+^ RGLs, an inverted ‘Y’ shape from anti-nestin staining superimposed on MCM2^+^ nucleus was scored double positive for nestin and MCM2, as described previously [45]. All analyses were performed by investigators blind to experimental conditions. Statistical significance (*p* < 0.01) was assessed with a one-way ANOVA or Student’s *t* test, as indicated.
